# Identificación de una variante en el gen *USH1G* en una familia con el síndrome de Usher

**DOI:** 10.7705/biomedica.7498

**Published:** 2025-09-22

**Authors:** Nancy Gélvez, Greizy López, Marta L. Tamayo

**Affiliations:** 1 Instituto de Genética Humana, Pontificia Universidad Javeriana, Bogotá, D. C., Colombia Pontificia Universidad Javeriana Instituto de Genética Humana Pontificia Universidad Javeriana Bogotá D. C Colombia

**Keywords:** síndromes de Usher, genética, retinitis pigmentosa, pérdida auditiva sensorineural, análisis mutacional de ADN, Colombia, Usher syndromes, genetics, retinitis pigmentosa, hearing loss, sensorineural, DNA mutational analysis, Colombia

## Abstract

El síndrome de Usher se caracteriza por hipoacusia neurosensorial congénita, retinitis pigmentaria y disfunción vestibular. Es la causa más frecuente de sordoceguera en el mundo. Se divide en tres tipos clínicos y doce subtipos genéticos. Se reporta el caso de una familia afectada por el síndrome de Usher debido a una variante del gen *USH1G* que codifica para la proteína SANS. Se realizaron los estudios clínicos oculares y auditivos correspondientes para la confirmación clínica del diagnóstico. El estudio molecular consistió en un panel de secuenciación de nueva generación que contenía 14 genes asociados con el síndrome de Usher: *MYO7A, USHC1, CDH23, PCDH15, USH1G, CIB2, USH2A, ADGRV1, WHRN, CLRN1, HARS, PDZD7, CEP250, C2orf71.* Se trata de una joven de 13 años, de una familia colombiana consanguínea, a quien se le diagnosticó un síndrome de Usher de tipo 1G. Las evaluaciones clínicas confirmaron las alteraciones auditivas, vestibulares y oculares y el análisis molecular identificó la variante homocigota p.Glu171Ter del gen *USH1G.*

Se resalta la importancia del diagnóstico temprano del síndrome de Usher. Aunque la frecuencia de variantes del gen *USH1G* es baja, no debe subestimarse; por el contrario, se recomienda su búsqueda activa para establecer la etiología exacta en esas familias. Se resalta la importancia de contar con un panel de variantes propias de la población colombiana para lograr diagnósticos más acertados y, en el futuro, buscar terapias génicas.

## Abstract (en)

Usher syndrome is characterized by congenital sensorineural hearing loss, retinitis pigmentosa, and vestibular dysfunction. It is the most common cause of deafblindness worldwide. It is classified into three clinical types and twelve genetic subtypes. We report a case of a family affected by Usher syndrome due to a variant in the *USH1G* gene, coding for the SANS protein. The ocular and auditory tests were performed for clinical confirmation of the diagnosis. The molecular study consisted of a next-generation sequencing panel containing 14 genes associated with Usher syndrome: *MYO7A, USHC1, CDH23, PCDH15, USHG1, CIB2, USH2A, ADGRV1, WHRN, CLRN1, HARS, PDZD7, CEP250, C2orf71.*

We present the case of a 13-year-old girl from a consanguineous Colombian family diagnosed with Usher syndrome type 1G. Clinical evaluations confirmed auditory, vestibular, and ocular alterations. Molecular analysis identified the homozygous p.Glu171Ter variant in the *USH1G* gene.

We highlight the importance of an early diagnosis of Usher syndrome. Although the variant frequency in the *USH1G* gene is low, it should not be underestimated; the exact etiology must be identified in these families. We recommend establishing a panel with Colombian-specific variants to perform more accurate Usher syndrome diagnoses, and in the future, to guide the development of gene therapies.

En diferentes estudios poblacionales, el síndrome de Usher se ha reportado como la causa más común de sordoceguera, incluso en Colombia. Es una entidad muy heterogénea, tanto desde el punto de vista clínico como genético.

Se estima que la prevalencia del síndrome de Usher en Colombia es de 3,3 casos por 100.000 habitantes. Se determinó que el 9 % de los niños que asisten a escuelas para sordos tienen el síndrome, así como el 10 % de aquellos que asisten a escuelas para ciegos ^1^^,^^2^.

Existen tres tipos de síndrome de Usher. El tipo I (USH1 MIM#276900) es la forma más grave y se caracteriza por sordera profunda congénita -generalmente prelingual- alteración del equilibrio y retinitis pigmentaria de inicio temprano, casi siempre se presenta en la primera década de la vida; el tipo II (USH2 MIM#608400, MIM#605472 y MIM#611383) se caracteriza por sordera congénita de leve a profunda, no presenta alteración del equilibrio y la retinitis pigmentaria aparece a partir de la segunda década de la vida, y el tipo III (USH3 MIM#276902) es el más infrecuente y se caracteriza por hipoacusia progresiva, que puede cursar con disfunción vestibular o sin ella, y por retinitis pigmentaria con una edad variable de aparición. Los tres tipos clínicos del síndrome de Usher tienen un mecanismo de herencia autosómico recesivo ^3^^,^^4^.

El síndrome de Usher de tipo I es una ciliopatía poco frecuente. La retinitis pigmentaria se traduce en disminución de la visión por alteración de la agudeza visual, disminución del campo visual y ceguera nocturna o nictalopía. El daño vestibular suele manifestarse con retraso del desarrollo motor o pérdida del equilibrio ^5^.

De los tres tipos descritos, el tipo I es causado por variantes en diversos genes, tales como: *MYO7A, USH1C, CDH23, PCDH15*y *USH1G*^6^^-^^9^.

El gen *USH1G* se encuentra en el locus 17q24-25 10, tiene 7,2 kb y tres exones, dos de ellos codificantes. El marco abierto de lectura de 1.380 pb codifica para una proteína de 460 aminoácidos denominada SANS (proteína de andamiaje con repeticiones de anquirina y un dominio SAM) ^11^. Hasta la fecha, solo se han documentado 18 variantes patógenas en este gen (http://deafnessvariationdatabase.org), la mayoría de ellas conducen a un fenotipo típico de USHI ^12^^,^^13^. En otras publicaciones se han reportado tres pequeñas deleciones ^14^, una de ellas asociada con un fenotipo similar al del tipo 2 ^15^.

El objetivo de este estudio fue analizar un caso infrecuente del síndrome de Usher de tipo I para establecer las variantes presentes en el gen *USH1G* y describir el cuadro clínico de la paciente portadora de una alteración en la proteína SANS.

## Presentación de caso

Se trata de una joven de 13 años, detectada gracias al proyecto de tamizaje nacional en instituciones para sordos que se adelanta desde el Instituto de Genética Humana de la Pontificia Universidad Javeriana. El padre y la madre de la menor también fueron incluidos en el estudio.

El diagnóstico clínico de síndrome de Usher de tipo I se basó en los criterios clínicos y genéticos establecidos ^2^. La joven fue diagnosticada según su historia clínica, evolución, y exámenes audiológico y oftalmológico. La paciente presentaba hipoacusia profunda, alteración del equilibrio, y signos y síntomas de retinitis pigmentaria. La pérdida auditiva se confirmó mediante audiometría tonal pura. Sus pruebas de equilibrio resultaron alteradas y su fenotipo retiniano se obtuvo mediante examen de fondo de ojo, angiografía y campimetría.

### Estudio genético

Previa obtención del asentimiento y consentimiento informado, a la paciente se le tomó muestra de sangre mediante punción de vena periférica para la extracción de ADN genómico con fenol-cloroformo.

Se utilizó un panel de secuenciación de nueva generación que contenía 14 genes asociados con el síndrome de Usher: *MYO7A*, *USHC1*, *CDH23*, *PCDH15*, *USHG1*, *CIB2*, *USH2A*, *ADGRV1*, *WHRN*, *CLRN1*, *HARS*, *PDZD7*, *CEP250*, *C2orf71*. La secuenciación se realizó en la plataforma Ion Torrent (Ion PGMTM System, Thermo Scientific). Las variantes sospechosas de ser patógenas se validaron por secuenciación Sanger.

### Reporte de caso

**Figura 1:**
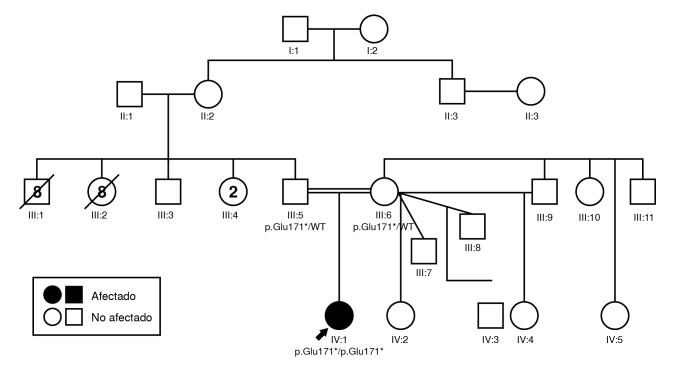
Árbol genealógico de la familia afectada por mutaciones en el gen *USH1G*

Se presenta el caso de una joven con diagnóstico de síndrome de Usher de tipo 1. Los padres son primos en primer grado de consanguinidad (figura 1). La paciente 40USH tenía 13 años en el momento de su inclusión en el estudio. Presentaba antecedentes de retraso en el desarrollo psicomotor, hipoacusia congénita e inicio de retinitis pigmentaria en la primera década de la vida.

Círculo: mujeres; cuadrado: hombres; símbolos vacíos: sin afectación; símbolos sólidos: individuos afectados; símbolos tachados: fallecidos; número dentro del símbolo: número de hermanos del mismo sexo

Los resultados de la audiometria mostraron sordera neurosensorial congénita bilateral profunda, no progresiva. Los potenciales auditivos del tallo cerebral se encontraban alterados, ya que no hubo respuesta. No se identificaron los potenciales I, II, III, IV y V cuando se usaron umbrales desde 50 a 100 dB. El timpanograma reveló una onda normal de tipo A en el oído derecho y alteración de la respuesta en el oído izquierdo.

Respecto a los resultados del examen oftalmológico, la paciente presentó disminución de la agudeza visual y de la visión en la primera década de la vida, con diagnóstico de retinitis pigmentaria. Se evidenció una alteración moderada del calibre de los vasos retiñíanos, pigmentación con apariencia de escasas espículas de hueso y alteración del epitelio pigmentario de la retina en media periferia, sin que en ese momento se evidenciara palidez del nervio óptico. Con estos hallazgos, se confirmó el diagnóstico de retinitis pigmentaria típica.

En los antecedentes familiares, la joven afectada es hija única. Su padre no presentaba alteraciones visuales o auditivas, mientras que su madre tenía una alteración visual leve, no definida. Una de sus abuelas y sus tías manifestaron "no ver bien" y tenían diagnóstico de ceguera sin causa conocida, posiblemente por catarata senil. El resto de sus familiares no presentaba sintomatologia relacionada con sordoceguera. Otros familiares ya habían fallecido (figura 1).

### Análisis molecular

Los resultados del análisis molecular se presentan en el cuadro 1. Se identificó la variante patógena homocigota c.511G>T, p.Glu171Ter en el gen *USH1G,* la cual no se había reportado previamente. La variante se identificó en ambos padres, cada uno heterocigoto y portador sano.

**Cuadro 1:** Variante identificada en el gen *USH1G*

Gen	Transcrito	Variante	Tipo	Cigosidad	Clasificación
*USH1G*	NM_173477.5	c.511G>T,	*Nonsense*	Homocigota	Patógena
(*SANS*)		p.Glu171Ter	(Sin sentido)		

### Consideraciones éticas

Este estudio fue aprobado por el Comité de Investigación y Ética de la Facultad de Medicina de la Pontificia Universidad Javeriana. Todos los individuos incluidos firmaron el consentimiento informado en el que aceptaban la recolección y el tratamiento de las muestras y los datos de la historia clínica, y la publicación del caso.

## Discusión

En este reporte se presenta el caso de una familia con diagnóstico de síndrome de Usher 1G por una variante patógena del gen *USH1G,* detectada en el programa de tamizaje para familias con el síndrome en instituciones para personas ciegas en Colombia.

El síndrome de Usher es la causa más frecuente de sordoceguera en el mundo ^16^. La prevalencia del síndrome de Usher se ha calculado entre 3,5 a 6,2 casos por cada 100.000 habitantes ^1^^,^^2^. Su frecuencia en Estados Unidos es de alrededor 5 casos por cada 100.000 habitantes y en Escandinavia es de 3 casos por cada 100.000 ^17^^,^^18^. Como ya se mencionó, en Colombia su prevalencia es de 3,3 por cada 100.000 habitantes y corresponde al 9,6% de la población sorda y al 10% de la población ciega ^1^. El síndrome de Usher tiene una heterogeneidad genética significativa, ya que a cada uno de sus tres tipos clínicos le corresponde uno o varios subtipos genéticos.

El síndrome de Usher es una enfermedad genética, de herencia autosómica recesiva, que cursa con hipoacusia neurosensorial, retinitis pigmentosa progresiva y disfunción vestibular. Clínicamente, se clasifica en los tres tipos antes descritos.

El presente caso se trata de una hija única con sordoceguera, con diagnóstico clínico de síndrome de Usher de tipo 1. El diagnóstico molecular se efectuó mediante secuenciación de nueva generación en la plataforma Ion Torrent, utilizando un panel de 14 genes asociados con dicho síndrome. Posteriormente, las variantes identificadas se validaron mediante secuenciación Sanger. Los análisis moleculares revelaron una variante patógena en el gen *USH1G* y permitieron excluir los demás loci USH conocidos.

El gen *USH1G* abarca más de 7,2 kb, contiene tres exones, de los cuales dos codifican una proteína de andamiaje expresada en la cóclea (incluidas las células ciliadas internas y externas), el aparato vestibular, la retina, el cerebelo y los testículos ^19^.

La confirmación de este caso es relevante para enfatizar varios aspectos en la atención de la población infantil que padece este tipo de enfermedades tan poco frecuentes. En primer lugar, si bien el síndrome de Usher es la causa principal de sordera y ceguera a nivel mundial, con frecuencia se olvida o rara vez se considera, especialmente, en niños que presentan solo sordera a una edad muy temprana. Por esta razón, es imperioso hacer un seguimiento oftalmológico en estos casos, ya que más adelante podrían aparecer signos y síntomas oculares que impacten el diagnóstico y el pronóstico en ellos.

En segundo lugar, se resalta la importancia de un diagnóstico temprano para detectar oportunamente alteraciones auditivas y visuales. Para mejorar el desarrollo del lenguaje y la comunicación, la sordera debe detectarse antes de los dos años de edad. El manejo adecuado de la pérdida auditiva en un niño es crucial para su desarrollo físico y mental, en especial, si se documentan alteraciones del equilibrio. Asimismo, el diagnóstico oftalmológico temprano en la población infantil es fundamental para el pronóstico visual, ya que algunas cegueras congénitas y otras alteraciones se manifiestan tardíamente; muchas veces, la pérdida visual es lenta pero progresiva. Sin una vigilancia adecuada, pueden transcurrir muchos años antes de que un niño refiera dificultades para ver en general o en condiciones de penumbra u oscuridad.

En tercer lugar, debería establecerse como parte de un protocolo obligatorio que en todo niño con sordera -ya sea congénita, genética o adquirida- se evalúen periódicamente el equilibrio y la visión.

En cuarto lugar, en caso de considerarse la posibilidad diagnóstica del síndrome de Usher, es usual que no se tenga en cuenta y evalúe la posible presencia en el gen *USH1G (SANS),* dada su escasa frecuencia. A nivel mundial, el subtipo USH1G es raro (0 a 4 %), pero no por ello debe desestimarse ni subestimarse (cuadro 2).

Por último, es importante resaltar las implicaciones éticas que conlleva el diagnóstico genético de enfermedades raras, como el síndrome de Usher. La identificación de una variante patógena homocigota requiere especial atención durante la asesoría genética familiar, ya que no solo impacta a nivel emocional, sino también, en la toma de decisiones. Es imperativo garantizar la confidencialidad de la información, ya que es un derecho fundamental de todo individuo involucrado.

**Cuadro 2:** Frecuencia del gen *USH1G* a nivel mundial

Gen	Subtipo de USH1	USH1 atribuible a variantes patógenas (%)	Proporción de variantes patógenas por método	
			Análisis de secuencia	Deleciones o duplicaciones
*MYO7A*	USH1B	53-70	~ 98 %	< 2 %
*USH1C*	USH1C	6-15	> 98 %	2 reportes
*CDH23*	USH1D	10-20	~ 85 %	< 15 %
*PCDH15*	USH1F	7-12	~ 75 %	~ 25 %
*USH1G*	USH1G	0-4 (raro)	> 85 %	2 reportes
*CIB2*	USH1J	Desconocido	1 reporte	Ninguno reportado
Desconocido	--	10-15		N/A

USH1: síndrome de Usher de tipo 1

Fuente: https://www.ncbi.nlm.nih.gov/books/NBK1265/

De igual manera, se debe considerar el impacto psicosocial que conlleva el diagnóstico de este tipo de enfermedades, pues puede generar ansiedad, sentimientos de culpa en los padres portadores y preocupaciones sobre el futuro de la persona afectada. Por ello, el acompañamiento psicoemocional debe ser una parte integral del proceso de asesoría.

Por otro lado, es esencial ofrecer a las familias alternativas relacionadas con los riesgos reproductivos, como opciones de diagnóstico prenatal o preimplantacional, así como la posibilidad del diagnóstico temprano en otros miembros de la familia. Todos estos aspectos se vuelven más relevantes cuando el acceso a los servicios de salud, específicamente los genéticos, es limitado. En este sentido, la atención clínica y genética de estos casos debe desarrollarse dentro de un sólido marco bioético, considerando el contexto sociocultural particular de cada caso y garantizando la protección de los derechos, la autonomía y el bienestar de los pacientes y sus familias.

En conclusión, en este reporte se resalta la importancia de considerar el diagnóstico de Usher de tipo I en la población sorda infantil y juvenil. Aunque la frecuencia de variantes en el gen *USH1G* sea baja, no debe subestimarse, ya que su identificación permite aclarar la etiología en esas familias. Además, las variantes en el *USH1G* se han asociado a casos con grave afectación, como el aquí presentado. Se destaca, también, la necesidad de desarrollar un panel de variantes características de la población colombiana para lograr diagnósticos más acertados del síndrome de Usher.

A futuro, se requieren estudios de expresión que permitan establecer la naturaleza de las variantes genéticas de cada individuo y aporten a la búsqueda de terapias génicas que mitiguen el impacto de estas enfermedades poco frecuentes.
